# Classification of Foot-and-Mouth and Lumpy Skin Disease in Cattle Using Frozen Pretrained Encoders: A Comparison of Convolutional and Transformer Architectures

**DOI:** 10.3390/vetsci13090962

**Published:** 2026-09-14

**Authors:** Paulo Valerio, Wilfredo Ticona

**Affiliations:** Facultad de Ingeniería, Universidad Tecnológica del Perú, Lima 15072, Peru; u18308140@utp.edu.pe

**Keywords:** bovine health, Brier skill score, data leakage, domain shift, explainable artificial intelligence, external validation, linear probing, shortcut learning

## Abstract

**Objective:** To determine whether the architectural family of a pretrained encoder, convolutional or attentional, governs its transferability and the reliability of its probabilities under frozen transfer, and how much of the task can be solved once lesion morphology is removed from the input. **Methods:** Photographs of cattle labelled healthy, foot-and-mouth disease or lumpy skin disease were split into a development set of 1277 from a public Kaggle collection and an external set of 985 from two Zenodo records. Six encoders, three convolutional and three vision transformers (ViT-B/16), were frozen and read through an identical linear head under a grouped five-fold split with five seeds. Discrimination and calibration were co-primary, and the external set was read once. **Results:** External balanced accuracy ran from 0.906 to 0.819, both extremes being ViT-B/16 encoders; after Holm correction, the three leading encoders, two attentional and one convolutional, were indistinguishable. The lowest of the six returned a Brier skill score of −0.030. A 4 × 4 thumbnail reached 0.527 against 0.333 for chance and recovered 0.650 of the foot-and-mouth cases. **Conclusions:** The pretraining recipe, not the convolutional–attentional distinction, ordered both criteria. Discrimination gave no warning of the loss of probabilistic skill, so a proper scoring rule must be reported beside it. External validation cannot expose a cue present in both collections that travels with the label, and low-information controls must accompany it.

## Simple Summary

Foot-and-mouth disease and lumpy skin disease are two of the most damaging illnesses of cattle, and both can be suspected based on how an animal looks. This has encouraged the idea of screening herds from photographs taken on a phone. We tested six ready-made image models on 2262 photographs, training only a small classifier on top of each and checking the result on a separate set of pictures the models had never seen. The models agreed with the labels most of the time, and five of the six identified every case of foot-and-mouth disease. We also found something that should temper confidence in numbers of this kind. When each photograph was shrunk to a grid of sixteen coloured blocks, in which no blister and no lump can possibly be seen, a simple classifier still recognised two thirds of the foot-and-mouth cases. Much of what looks like disease recognition is recognition of how the picture was taken. That is a warning about the data, not a screening tool. Photographic screening should prompt a veterinary examination and laboratory confirmation, but never replace them. Future work needs photographs taken according to a fixed protocol, animal identifiers, laboratory-confirmed labels and lesion outlines drawn by specialists.

## 1. Introduction

Lumpy skin disease (LSD) and foot-and-mouth disease (FMD) are trans-boundary infections of cattle, and FMD ranks among the diseases whose control would most advance the Sustainable Development Goals [1]. Like other animal diseases, they keep reaching new areas [2]. LSD has spread from an African endemic to a global epizootic, with over 41,000 outbreaks between 2005 and 2020 [3]. Morbidity varies from 3% to 85% worldwide, while mortality typically remains low, around 1–3%, so the loss is chronic [4]. The 2022 epidemic in India affected some 2.4 million cattle, killed over 110,000 and cost above USD 60 million [3]. FMD costs more: production losses and vaccination in endemic regions are estimated at USD 6.5–21 billion a year [5].

Neither disease can be confirmed by sight. LSD produces raised, well-defined skin nodules with fever and swollen lymph nodes [3,4]. FMD produces blisters in the mouth, on the feet, and on the teats—tongue, buccal mucosa, coronary band—so it is not a skin disease [6]. The blisters burst into sores that other vesicular infections also cause, so diagnosis rests on real-time RT-PCR or antigen ELISA on tissue from a fresh lesion [6]. Outbreak records suffer where reporting relies on voluntary farmer engagement: labels are inconsistent and data entry is error-prone [7]. In Ethiopia, smartphone reporting improved the accuracy, completeness and timeliness of cattle disease records over paper [8], though case entry still depends on the reporting user’s own clinical judgement in the field.

Image classification has been applied to this problem many times, and the reported accuracies are consistently high. Work on LSD spans both transfer learning from ImageNet-pretrained backbones and purpose-built architectures trained from scratch: a comparison across ten models [9], and individual pipelines built on MobileNetV2, Inception, extreme learning machines and purpose-built networks, reports 90.1% to 96.9% accuracy [10,11,12,13]; a YOLO-based detector has also been proposed [14]. Work on FMD images is scarcer; the clearest combines convolutional and Haralick features [15]. A parallel line predicts cattle disease from clinical, behavioural or epidemiological variables rather than images, reporting 81% to 98% accuracy [16,17,18,19]. Together these studies show that the task can be learned.

That a task can be learned does not mean the reported figures can be trusted. For instance, a recent lumpy-skin-disease grading study trains and evaluates on a single public Kaggle dataset and reports validation accuracy rather than performance on a held-out test set [13]. Leakage does most harm in exactly these conditions. Kapoor and Narayanan found leakage in at least 294 papers across 17 fields, in eight forms, usually inflating the reported results [20]. Collections gathered from the web are especially exposed: a photograph and a rotated or rescaled copy can end up on opposite sides of a random split. To our knowledge, no study in this literature checks its collection for duplicates.

A second limitation is what an architectural comparison can prove. Convolutional networks [21,22] and vision transformers [23,24] have been compared in medical imaging, with answers that vary by task [25]. Fine-tuning alters the representation while measuring it, so the ranking describes the encoder together with its training, not the encoder alone. Poyrazer et al. avoid this in retinal grading by leaving the weights untouched and fitting only a small head, limiting the comparison to what the representation already holds [26]. No equivalent protocol exists for cattle images, where backbone and training usually change together.

Accuracy is also an incomplete criterion. For triage, a predicted probability must mean what it says: among cases a model calls 80% likely, close to 80% should be correct, so a threshold can separate the animals the system may settle from those needing a veterinarian. Modern neural networks are overconfident [27], and temperature scaling corrects this on data like the training set. Whether the correction survives a change of camera and setting has been examined for retinal images [26] but not for cattle, where calibration is not reported.

Finally, a comparison should establish what the ranking rests on. A network can reach high benchmark accuracy on cues unrelated to the disease, provided they accompany the label [28]. Collections built one disease at a time are exposed, since each condition tends to be photographed in its own way. Saliency maps are the usual way of looking inside a model, and they are informative once the map is shown to change when the model changes [29,30].

The objective of this study was to determine whether the architectural family of a pretrained encoder, convolutional or attentional, governs its transferability and the reliability of its probabilities when it is used without fine-tuning to classify field photographs of cattle as healthy, FMD-affected or LSD-affected. Six pretrained backbones, three convolutional and three ViT-B/16, were frozen and read through an identical linear head on identical 224 × 224 inputs, so that any difference between the families comes from the representation and not from how it was adapted. Heads were fitted on 1277 photographs from a public Kaggle collection [31] and applied once, without refitting, to 985 images merged from two independent Zenodo records [32,33], both checked for duplicates beforehand. Discrimination and calibration were measured on equal terms on each cohort, with confidence intervals and corrected comparisons. Two further checks were run: the heads were retrained on images with the lesion removed, to see how much of the result survives it; and the saliency maps were recomputed with randomised weights, to confirm they respond to the model.

## 2. Materials and Methods

**Figure 1:**
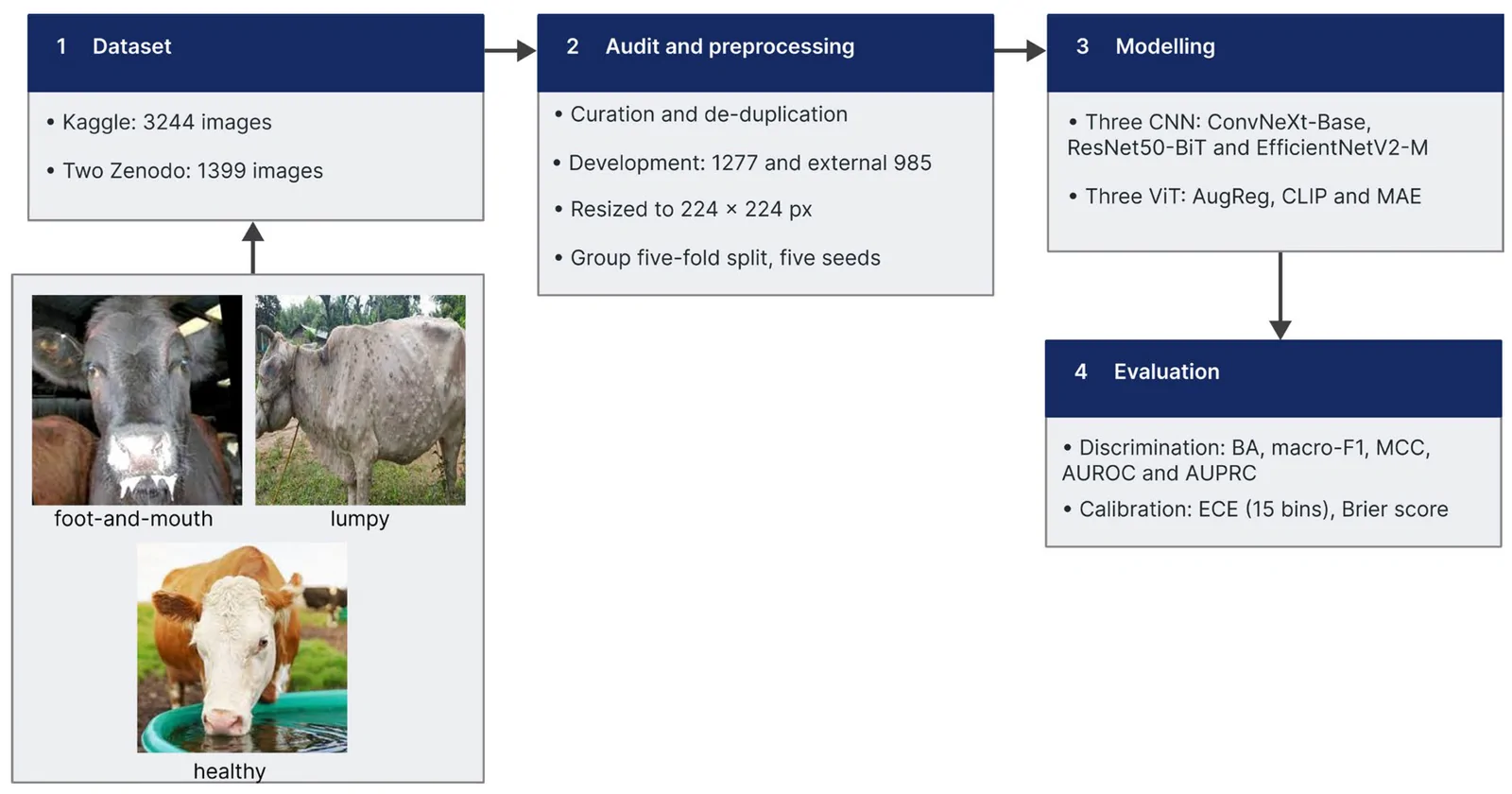
Methodology proposed.

Figure 1: The four phases of the study, with one example image per class. Phase 1 collects 3244 photographs from a public Kaggle collection and 1399 from two Zenodo records. Phase 2 curates and de-duplicates both, merges the two Zenodo records, and leaves 1277 development and 985 external images, read at 224 × 224 px and divided into five folds with five seeds, grouped so that near-duplicate images stay together. Phase 3 freezes three convolutional and three ViT-B/16 encoders and fits an identical linear head on each. Phase 4 reports discrimination and calibration, runs the shortcut controls and the saliency checks, and reads the external set once. BA, balanced accuracy; MCC, Matthews correlation coefficient; AUROC, area under the curve of the receiver operating characteristic; AUPRC, area under the curve of precision against recall; ECE, expected calibration error.

### 2.1. Dataset

#### 2.1.1. Kaggle (Development Set)

Cattle Diseases Datasets [31] is a public Kaggle collection of 3244 photographs distributed in three folders that carry the class label: healthy (*n* = 1291; 39.8%), lumpy skin disease (*n* = 1207; 37.2%) and foot-and-mouth disease (*n* = 746; 23.0%). Most images show cattle, though other species also appear. All but one file are JPEG, and sizes range from 153 to 4608 pixels wide across 245 distinct dimensions, of which 640 × 640 (35.4%) and 300 × 300 (24.1%) are the most common.

The file names indicate an aggregation of several sources. Some are camera or messaging exports (*n* = 368; 11.3%); some belong to breed-catalogue series covering Ayrshire, Brown Swiss, Holstein Friesian, Jersey and Red Dane cattle (*n* = 390; 12.0%); and others are sequentially numbered series (*n* = 926; 28.5%). Among those, 1849 files (57.0%) carry the token that Roboflow writes on export, so part of the collection passed through that annotation platform before publication.

#### 2.1.2. Zenodo (External Set)

Two independent Zenodo records make up the external set. The first, LumpySkinDisease_DataHub [32], is a single folder of 1019 PNG images, all of them 256 × 256 pixels, in which a prefix on the file name carries the label: normal skin (*n* = 697; 68.4%), mild lumpy skin disease (*n* = 231; 22.7%) and severe lumpy skin disease (*n* = 91; 8.9%). It is the only source used here that grades severity.

The second record, FMD Cattle [33], holds 380 files in two numbered folders, 173 for healthy animals (45.5%) and 207 for foot-and-mouth disease (54.5%). Of these, 342 are JPEG images, 324 of them (94.7%) at 512 × 512 pixels and the rest reaching 4160 pixels wide. The remaining 38 files, all in the disease folder, do not carry a standard image extension, and four of those cannot be decoded.

Its file names are clinical captions naming the region photographed, and the same regions recur under both labels: muzzle, open mouth, tongue, udder and teat, and foot or hoof. The most common are the dental pad (*n* = 51) and the tongue (*n* = 28) under the disease label, and the udder and the muzzle (*n* = 59 each) under the healthy label.

#### 2.1.3. Audit and Preprocessing

Both collections were audited before any model was fitted. Their folders held repeated photographs, augmented copies of images already present, images carrying a watermark, a logo or an overlaid caption, drawings and illustrations rather than photographs, images too degraded to show the animal, files that could not be decoded, and animals of species other than cattle.

Images were removed on those grounds. Repeated and near-repeated photographs were grouped by exact and perceptual image hashing and the groups were then confirmed by eye, one representative being kept; the remaining grounds were decided by inspection. The search was run at three scopes: within each class, between the classes of one collection, and between the development and the external collections, this last so that no photograph reaching the external set had a counterpart among the images used to fit the models.

After curation, the development set held 1277 images, healthy (*n* = 753; 59.0%), lumpy skin disease (*n* = 380; 29.8%) and foot-and-mouth disease (*n* = 144; 11.3%), and the external set held 985, healthy (*n* = 703; 71.4%), lumpy skin disease (*n* = 179; 18.2%) and foot-and-mouth disease (*n* = 103; 10.5%). Every retained image was converted to RGB and brought to a common resolution of 224 × 224 px (Table 1).

**Table 1:** Composition after the curation described in Section 2.1.3.

Set	*n*	Healthy	FMD	LSD	Imbalance	Role
Development (Kaggle)	1277	753	144	380	5.2:1	5-fold CV, 5 seeds
External (Zenodo)	985	703	103	179	6.8:1	Single-use, zero tuning

### 2.2. Frozen Backbone Models

Six encoders were compared, three convolutional and three attentional. None of them was fine-tuned; each was used as a fixed feature extractor.

#### 2.2.1. ConvNeXt-Base

ConvNeXt-Base [34] is a convolutional network rebuilt around design choices taken from vision transformers: large depthwise kernels, inverted bottlenecks, and far fewer normalisation and activation layers than earlier convolutional networks. It was pretrained on ImageNet-21k [35] and then fine-tuned on ImageNet-1k. It holds 87.6 million parameters, the vector it returns has 1024 dimensions, and its native resolution is 224 × 224 px.

#### 2.2.2. ResNet50-BiT

ResNet50-BiT [36] is a fifty-layer residual network [21] from the Big Transfer family, which replaces batch normalisation with group normalisation and standardises the convolutional weights, a combination its authors found to transfer better than the original design. It was pretrained on ImageNet-21k and then fine-tuned on ImageNet-1k. It holds 23.5 million parameters, fewer than any other encoder used here, yet the vector it returns has 2048 dimensions, more than any other; its native resolution is 448 × 448 px.

#### 2.2.3. EfficientNetV2-M

EfficientNetV2-M [37] extends the scaling rules of EfficientNet [22] with an architecture search that rewards training speed as well as accuracy, and with early-stage blocks that use ordinary convolutions in place of depthwise ones. It was pretrained on ImageNet-21k and then fine-tuned on ImageNet-1k. It holds 52.9 million parameters, the vector it returns has 1280 dimensions, and its native resolution is 384 × 384 px.

#### 2.2.4. ViT-B/16 AugReg

ViT-B/16 AugReg [38] is a Vision Transformer of base size: the image is cut into 16 × 16 patches, each patch is linearly embedded, and a stack of self-attention blocks processes the resulting sequence. It was pretrained on ImageNet-21k under a schedule of augmentation and regularisation tuned for these models [39], and then fine-tuned on ImageNet-1k. It holds 85.8 million parameters, the vector it returns has 768 dimensions, and its native resolution is 224 × 224 px.

#### 2.2.5. CLIP ViT-B/16

CLIP ViT-B/16 [40] shares the architecture of Section 2.2.4 but was trained on a different signal. In place of class labels it learned from some 400 million image–text pairs gathered from the web, each image drawn towards its own caption and away from the rest. No fine-tuning on ImageNet followed, and the text branch plays no part here. It holds 85.8 million parameters, the vector it returns has 768 dimensions, and its native resolution is 224 × 224 px.

#### 2.2.6. MAE ViT-B/16

MAE ViT-B/16 [41] is the third encoder built on that architecture and the only one trained without labels. Three quarters of the patches of each image were masked and the network learned to reconstruct the missing pixels; the decoder that performed that task was then discarded and only the encoder was kept. Pretraining used the ImageNet-1k images alone, with no subsequent fine-tuning. It holds 85.8 million parameters, the vector it returns has 768 dimensions, and its native resolution is 224 × 224 px.

### 2.3. Linear Classification Head

Each encoder was run in inference mode and its weights were never updated; the only parameters fitted were those of a head placed on the features it produced. Images entered every encoder at 224 × 224 px without a centre crop, normalised with the statistics under which that encoder had been pretrained.

The head is a single linear layer mapping the embedding to the three classes. Ahead of it the features were standardised to zero mean and unit variance, the standardising statistics being estimated on the training fold alone. The loss was cross-entropy weighted by inverse class frequency, recomputed for every fold, which off-sets the imbalance reported in Section 2.1.3.

Optimisation used AdamW, with a learning rate of 1 × 10^−3^, a decay of 0.01 on the weights and a cosine annealing schedule, in batches of 64 for at most 100 epochs. Training stopped early once the macro-averaged F1 on the inner validation split had gone ten epochs without improving.

### 2.4. Evaluation Metrics

Two families of metrics were computed. Discrimination was measured by balanced accuracy, macro-averaged F1, the Matthews correlation coefficient (MCC), and two areas under curves, AUROC for the receiver operating characteristic and AUPRC for precision against recall, each computed one class against the rest and averaged over the three. Reliability, meaning whether the output probabilities can be read as probabilities, rests on three quantities: Brier score, Brier skill score (BSS) and expected calibration error (ECE), each reported on the raw probabilities and again after temperature scaling [27]. Every head fitted its own temperature on validation images held back inside its fold, and those values travelled to the external set untouched, so no calibration parameter was estimated where it was later judged.

With *K* = 3 for the classes and *n* for the images, and TP*_k_*, FP*_k_* and FN*_k_* as the true positives, false positives and false negatives of class *k*, respectively, Equations (1)–(4) define per-class recall and precision and the two macro measures built on them.

(1) $$R_{k}=\frac{{\mathrm{TP}}_{k}}{{\mathrm{TP}}_{k}+{\mathrm{FN}}_{k}}$$

(2) $$P_{k}=\frac{{\mathrm{TP}}_{k}}{{\mathrm{TP}}_{k}+{\mathrm{FP}}_{k}}$$

(3) $$BA=\frac{1}{K}\sum_{k=1}^{K}R_{k}$$

(4) $$\text{macro-F1}=\frac{1}{K}\sum_{k=1}^{K}\frac{2P_{k}R_{k}}{P_{k}+R_{k}}$$

Balanced accuracy replaces plain accuracy because the classes are unequal in size, and it gives every class the same weight whatever its prevalence. Equation (5) compresses the whole confusion matrix into one coefficient, the MCC, with c defined as how many predictions were correct, *t_k_* as how many images truly belong to class *k*, and *p_k_* as how many were assigned to it.

(5) $$\mathrm{MCC}=\frac{\mathrm{cn}-\sum_{k}p_{k}t_{k}}{\sqrt{\left(n^{2}-\sum_{k}p_{k}^{2}\right)\left(n^{2}-\sum_{k}t_{k}^{2}\right)}}$$

The AUROC gives the chance that an image of one class outranks an image drawn from the others; the AUPRC integrates precision against recall over that comparison and is the more informative of the two when the positive class is scarce, as foot-and-mouth disease is in both sets.

For the ECE, predictions are sorted into M bins of equal width by the confidence of the class they name; *B_m_* holds those in bin m, and acc (*B_m_*) and conf (*B_m_*) are the share of them classified correctly and their mean confidence. Equation (7) squares the gap between the probability vector *p_i_* and the one-hot label *y_i_*, sums it over the classes and averages over the images, so it runs from 0 to 2. Equation (8) sets that against BS_ref_, the same quantity for a forecast that always returns the class prevalences of the set being judged:

(6) $$\mathrm{ECE}\;=\sum_{m=1}^{M}\frac{\left|B_{m}\right|}{n}\cdot \left|\mathrm{acc}\left(B_{m}\right)-\mathrm{conf}\left(B_{m}\right)\right|,\;M=15$$

(7) $$\mathrm{BS}=\frac{1}{n}\;\sum_{i=1}^{n}\sum_{k=1}^{K}{\left(p_{\mathrm{ik}}-y_{\mathrm{ik}}\right)}^{2}$$

(8) $$\mathrm{BSS}=1-\frac{\mathrm{BS}}{{\mathrm{BS}}_{\mathrm{ref}}}$$

Smaller ECE and Brier values mean better calibrated probabilities; for every other metric, larger is better. A BSS of zero says the probabilities carry no more information than the prevalences alone, and a negative BSS that they carry less. Because confidence cannot drop below 1/K = 0.333, the five lowest bins are necessarily empty and add nothing to the sum.

### 2.5. Evaluation Protocol

The development set was divided into five folds by a grouped, stratified split that preserved the class proportions and never broke apart a near-duplicate cluster of Section 2.1.3. The folds hold 256, 256, 255, 255 and 255 images, each containing 28 or 29 foot-and-mouth cases, and no cluster was divided between them. Inside the four training folds of every round, a further grouped split set aside about 146 images, used for early stopping and for fitting the temperature. Each round was repeated with five initialisation seeds, which gives 25 heads per encoder.

Two quantities were then measured. On the development set every image was scored by the heads that had never seen it and calibrated with the temperature of its own fold, so the figure is an out-of-fold estimate. On the external set, all 25 heads made a prediction and their probabilities were averaged, with the spread across the five folds reported separately as a measure of dispersion. The order of operations is fixed throughout: logits are divided by the temperature of the head that produced them, turned into probabilities, and only then averaged. Averaging the logits instead would pool them geometrically and yield a different, more confident quantity.

One encoder serves as the reference against which the others are compared. It was chosen on development balanced accuracy alone, and that choice was written to disk with a timestamp before any external metric had been computed. The external set itself was read once, after every decision taken on the development set had been fixed.

The whole procedure was then repeated under four variants, to check that the comparison does not rest on a single design decision: a head carrying a 512-unit hidden layer, an unweighted loss, the features reduced to 512 components by principal component analysis, and folds drawn at random instead of by cluster.

### 2.6. Statistical Analysis

Every comparison was made against the reference encoder on the external set, over the same images, using a paired bootstrap of 10,000 stratified resamples with the significance level set at 0.05.

The tests were declared in two families before any of them was run. The confirmatory family pits the reference against each of the five rivals on balanced accuracy and on macro-averaged F1, ten tests in all, and its *p*-values are corrected by the Holm procedure. The exploratory family covers per-class recall and the mean Brier score, with the Benjamini–Hochberg procedure applied inside each subfamily rather than across the whole set. Intervals come from the percentile method for quantities read off the confusion matrix, and from the bias-corrected and accelerated method for the Brier mean, whose statistic is an average over individual images.

McNemar’s test accompanies the bootstrap on the same pairs. The two answer different questions and may therefore disagree: McNemar counts every image on which the two models differ, false positives included, whereas a bootstrap on the recall of one class only ever sees the images that belong to it. Both are reported so that the discrepancy is visible rather than resolved by choosing one.

As a sensitivity check, the whole procedure was repeated by resampling near-duplicate clusters instead of images. The mean design effect was 1.02, so the residual dependence left by the audit of Section 2.1.3 is negligible and the two schemes give the same conclusions.

### 2.7. Shortcut Control Experiments

Five deliberately impoverished descriptors were built from the same images as a control: the mean and standard deviation of each colour channel, a sixteen-bin colour histogram, and thumbnails of 4 × 4, 8 × 8 and 16 × 16 pixels. None keeps enough detail to show a vesicle or a nodule, and the largest has as many dimensions as a ViT-B/16 embedding.

Each descriptor passed through the head, the folds, the seeds and the calibration of Section 2.3, Section 2.4 and Section 2.5 unchanged, so that any gap in performance is attributable to the input and not to the procedure; the equivalence was confirmed by running a real encoder’s features through the same code and recovering its external balanced accuracy exactly. A floor was obtained by shuffling the training labels while leaving the test labels intact, and the distance between the descriptors and the full models was estimated with the paired bootstrap of Section 2.6.

### 2.8. Explainable AI

Saliency maps were computed for the images of the external set that all six encoders classified correctly and on which the ensemble held a minimum margin, so that a comparison between encoders is not confounded by disagreement over the prediction itself. For the three convolutional encoders, the map is Grad-CAM taken over the last spatial representation; for the three transformers it is the gradient–activation product at the input of the last block, with LeGrad computed alongside it as a robustness variant.

These methods were then put through the randomisation checks of Adebayo et al. [29]. The first replaces the weights of the linear head with random values, on 24 images; the second randomises the backbone in cascade, block by block, on 12. Each resulting map is compared with the original by correlation and read between two references: a ceiling, given by the agreement between maps produced by two halves of the 25 heads, and a floor, given by the agreement with the map of a different image of the same class. A map whose correlation stays near the ceiling after randomisation is not responding to the model that produced it.

### 2.9. Software and Hardware

Everything was implemented in Python 3.13.15 with PyTorch 2.11.0, timm 1.0.28, NumPy 2.1.3 and pandas 2.2.3; scikit-learn 1.6.1 provided the partitions and the standardisation, SciPy 1.16.3 the correlation statistics, and ImageHash 4.3.2 the perceptual hashes. The six pretrained encoders were downloaded through timm itself, and all computations ran in Google Colab (Google LLC, Mountain View, CA, USA) on a single NVIDIA L4 graphics processor (NVIDIA Corporation, Santa Clara, CA, USA).

## 3. Results

### 3.1. Discrimination

CLIP ViT-B/16 reached the highest balanced accuracy on both sets, 0.913 on development and 0.906 externally, and MAE ViT-B/16 the lowest, 0.868 and 0.819 (Table 2). Both ends of the ranking therefore belong to the same architecture, since the three ViT-B/16 encoders share the backbone of Section 2.2.4 and differ only in how they were pretrained. The three convolutional encoders fall between them, from 0.882 to 0.872 on the external set.

Moving to the external set cost every encoder something, but not equally: the loss in balanced accuracy ran from 0.003 for EfficientNetV2-M and 0.006 for CLIP ViT-B/16 to 0.049 for MAE ViT-B/16. Ordering the six by macro-F1 or by the MCC returns the same positions as balanced accuracy, while the AUROC compresses them into a band from 0.948 to 0.978.

**Figure 2:**
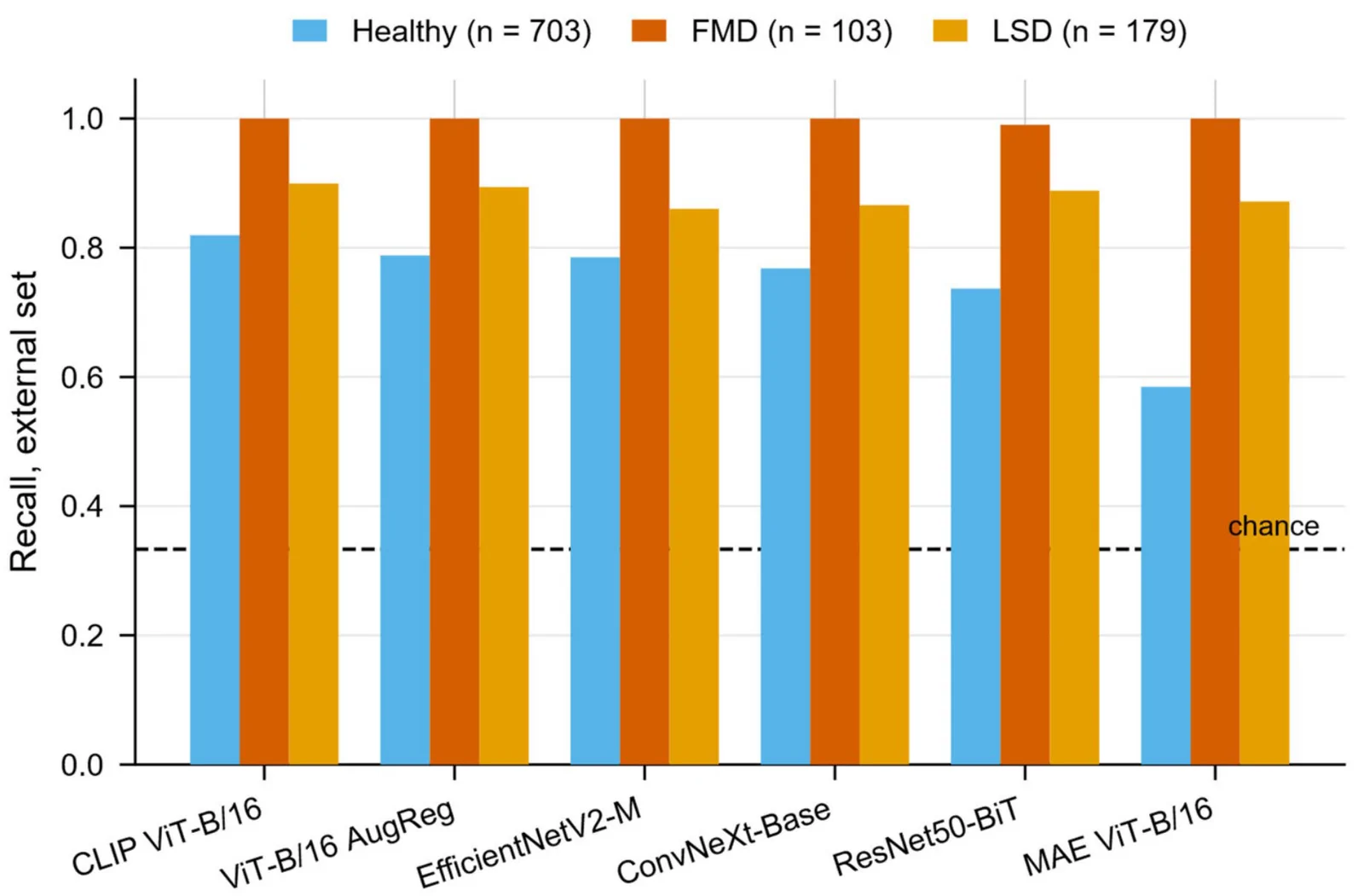
Per-class recall on the external set. The dashed line marks chance for three classes.

The per-class picture is uneven (Figure 2). Foot-and-mouth disease was recovered in full by five of the six encoders and in 102 of its 103 images by the sixth. Lumpy skin disease followed, between 0.860 and 0.899. The healthy class was the weakest for every encoder, from 0.819 in CLIP ViT-B/16 down to 0.585 in MAE ViT-B/16, and it carries most of the distance between the two ends of the ranking: those two encoders differ by 0.235 in healthy recall and by 0.028 in lumpy skin disease.

### 3.2. Calibration

Development calibration was good for every encoder, with the calibration error lying between 0.019 and 0.049 and the Brier skill score between 0.622 and 0.747 (Table 3). The external set separated them. Five stayed between 0.022 and 0.040, while MAE ViT-B/16 rose to 0.101 and its skill score fell to −0.030, meaning its probabilities carried less information than always answering the class prevalences of the set, even though it classified 0.819 of the images correctly by balanced accuracy.

Brier scores are not comparable between the two sets on their own, because the external set is the more imbalanced and its irreducible uncertainty is 0.104 lower; each skill score in Table 3 is referred to the climatological forecast of its own set.

**Figure 3:**
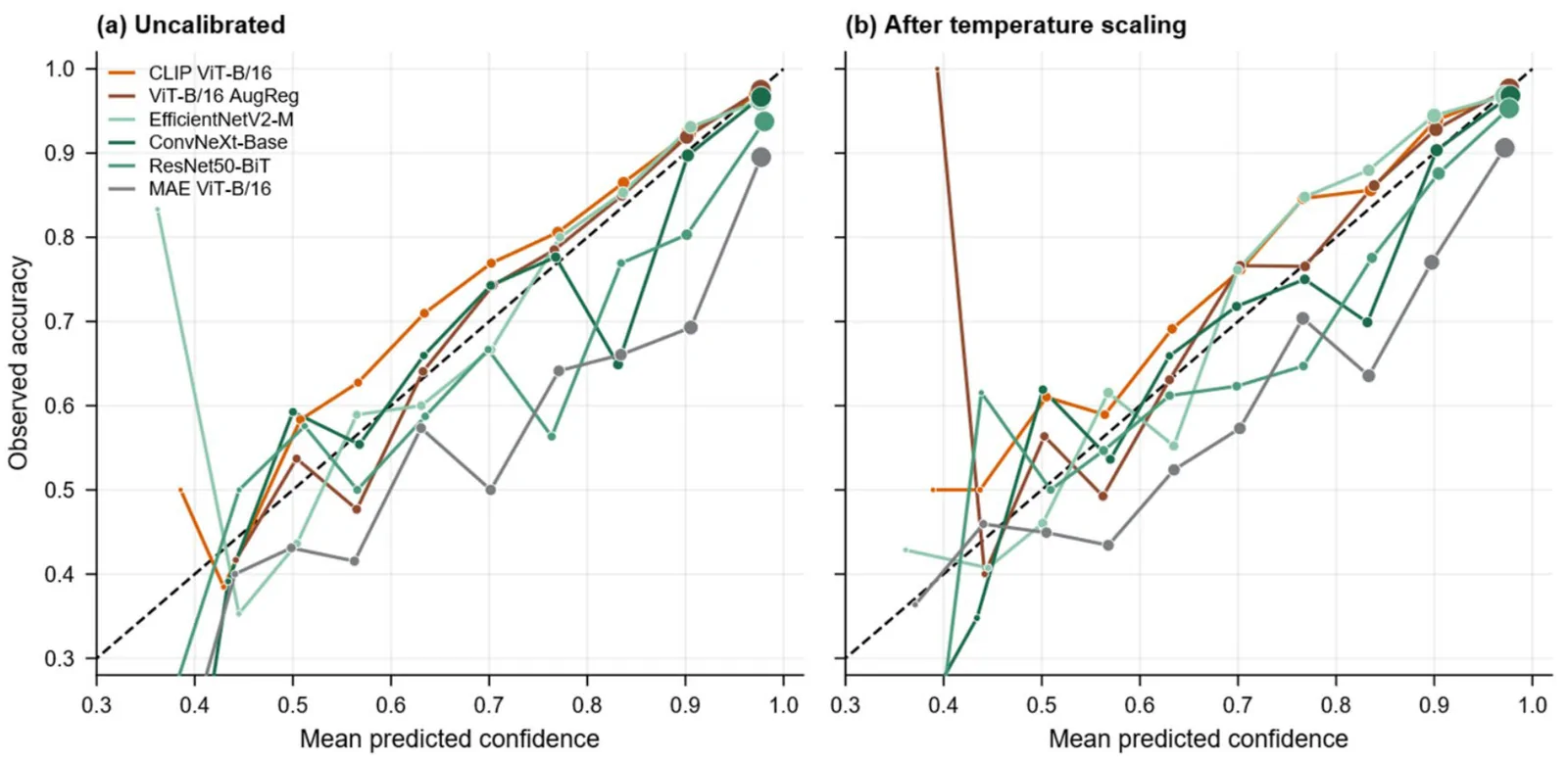
Reliability on the external set, before and after temperature scaling. The dashed line is perfect calibration; a curve below it belongs to a model that promises more than it delivers. Marker area is proportional to the number of images in the bin. Bins below 1/K = 0.333 are structurally unreachable.

The fitted temperatures ran from 1.045 to 1.243, so every head was mildly overconfident, and scaling by them barely moved external calibration: three encoders improved and three worsened, all by small margins. Figure 3 shows the shape behind those numbers. Before scaling, CLIP ViT-B/16 and ViT-B/16 AugReg lie above the diagonal over most of the range, whereas MAE ViT-B/16 lies below it throughout, promising more than it delivers; after scaling, the curves move little.

Refitting a per-class vector of scales on development data lowered external calibration error for all six encoders, most sharply for MAE ViT-B/16, where it fell from 0.122 to 0.040 against a single temperature fitted on the same pooled probabilities. The residual miscalibration is therefore class-dependent and beyond the reach of one scalar.

### 3.3. Statistical Comparison

CLIP ViT-B/16 served as the reference, fixed on development balanced accuracy before any external metric was computed (Section 2.5). Six of the ten confirmatory tests survived the Holm correction (Table 4). The reference beat ConvNeXt-Base, ResNet50-BiT and MAE ViT-B/16 on both balanced accuracy and macro-F1, and separated from neither EfficientNetV2-M nor ViT-B/16 AugReg on either metric, the corrected *p*-values there running from 0.062 to 0.185 and both intervals for AugReg covering zero. The three encoders that cannot be told apart are two transformers and one convolutional network.

In the exploratory family, corrected inside each subfamily, the differences concentrated in one class. Recall of the healthy class favoured the reference over four of the five comparators, by 0.034 to 0.235, with q at most 0.043, the exception being ViT-B/16 AugReg at q = 0.074. Recall of lumpy skin disease gave no significant difference against anyone. The mean Brier score favoured the reference over ConvNeXt-Base, ResNet50-BiT and MAE ViT-B/16, at q = 0.0003 in each case, and over neither of the other two.

Foot-and-mouth disease is where the two tests part company. Five encoders recovered every one of its 103 images, so the difference in recall is exactly zero against four comparators and the bootstrap returns q = 1.000 against all five. McNemar, computed one class against the rest, rejects for four of the five, with *p* from below 0.0001 to 0.048. The two are not in conflict: recall counts only the images that belong to the class, whereas McNemar counts every image on which two models disagree, including those wrongly assigned to it. Both are reported.

### 3.4. Shortcut Controls

**Figure 4:**
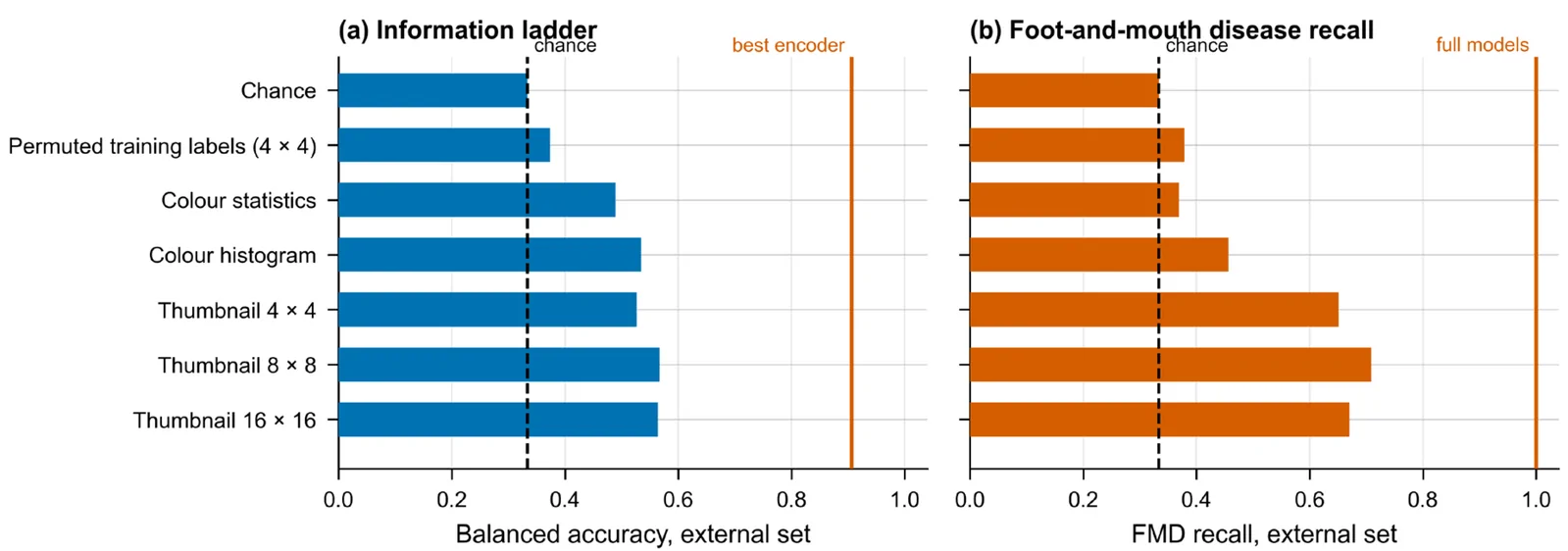
Information ladder. (**a**) External balanced accuracy of each impoverished descriptor; (**b**) external recall of foot-and-mouth disease for the same descriptors. Vertical lines mark the full models.

Shuffling the training labels left the pipeline at 0.374 balanced accuracy on the external set, against 0.333 for chance, so the procedure of Section 2.3, Section 2.4 and Section 2.5 does not manufacture a signal of its own (Figure 4).

The impoverished descriptors did considerably better than that. Six numbers, the mean and standard deviation of each colour channel, reached 0.489. A colour histogram reached 0.535, a 4 × 4 thumbnail 0.527 and an 8 × 8 thumbnail 0.567. Going up to 16 × 16, which yields the same 768 dimensions as a ViT-B/16 embedding, gained nothing over 8 × 8, at 0.564.

The three classes did not respond similarly. Colour statistics alone recovered 0.670 of the lumpy skin disease images but only 0.369 of the foot-and-mouth ones, barely above chance; the thumbnails reversed that, the 8 × 8 reaching 0.709 on foot-and-mouth disease and 0.503 on lumpy skin disease. Recall of the healthy class stayed between 0.428 and 0.522 for every descriptor.

The distance between descriptors and encoders was estimated with the paired bootstrap of Section 2.6, taking the 4 × 4 thumbnail as the comparator. In balanced accuracy, it ran from 0.292 for MAE ViT-B/16 to 0.380 for CLIP ViT-B/16, and in recall of foot-and-mouth disease it was 0.350 for five encoders and 0.340 for the sixth. All twelve comparisons returned *p* = 0.0002.

### 3.5. Saliency and Its Sanity Checks

**Figure 5:**
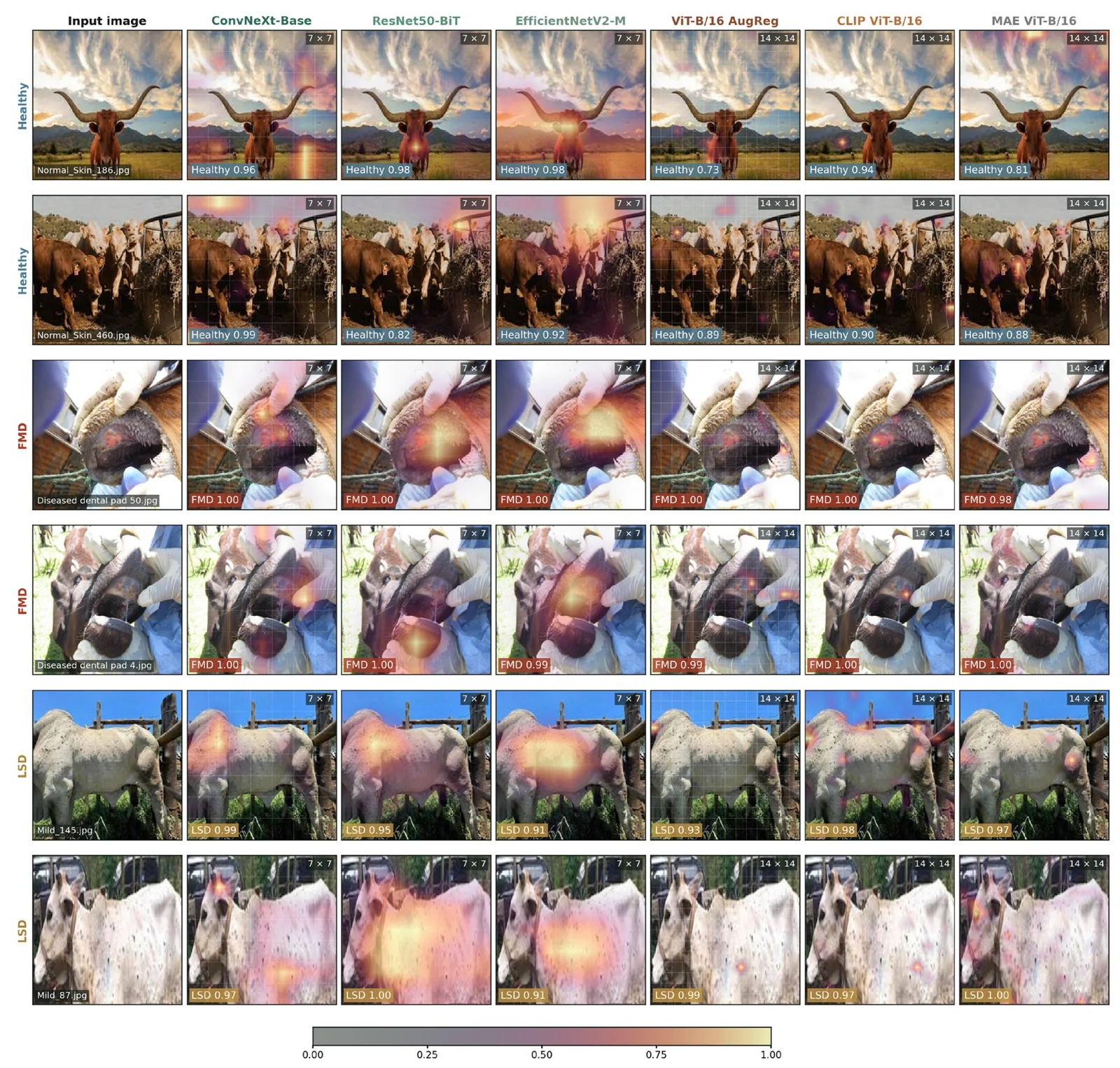
Saliency maps for six external images, two per class, across the six encoders. The tag gives the spatial resolution of the map; maps are relative within each panel and intensities are not comparable across panels.

Figure 5 shows the maps for six external images, two per class, drawn from those that all six encoders classified correctly and whose ensemble margin was closest to the median of their class.

Randomising the linear head collapsed every map. Over 24 images, the correlation with the original fell to between 0.025 and 0.191, against a ceiling of 0.950 to 0.983 taken from two halves of the 25 heads. For ResNet50-BiT and EfficientNetV2-M, it fell below the floor as well, that is, below the correlation between a map and the map of a different image of the same class.

Randomising the backbone in cascade did the same. With the last block alone randomised, the correlation lay between −0.070 and 0.105 for all six encoders, and that single block was enough to change the predicted class in 4 to 8 of the 12 images. For EfficientNetV2-M, the cascade returned an empty map in 8 of the 12, so its full-cascade value is not defined.

LeGrad behaved differently. With only the last block randomised, its maps held a correlation of 0.958 for ViT-B/16 AugReg, 0.938 for CLIP ViT-B/16 and 0.957 for MAE ViT-B/16, barely moving, although the predicted class had already changed in 8, 4 and 6 of the 12 images, respectively. Under the full cascade, LeGrad did decorrelate, to 0.003, −0.060 and 0.122. The maps in Figure 5 are accordingly the gradient–activation product and not LeGrad.

### 3.6. Robustness

Repeating the whole procedure under the four variants of Section 2.5 left the ranking of the six encoders unchanged in every case. A head carrying a 512-unit hidden layer raised validation macro-F1 by about 0.02 for all six, but its fitted temperatures rose from the 1.045 to 1.243 of Table 3 into the range 1.51 to 1.83, so the extra capacity bought accuracy at the price of confidence. Dropping the class weights from the loss lowered validation macro-F1 by 0.002 on average and left the temperatures almost where they were. Reducing the features to 512 principal components cost 0.005. Drawing the folds at random rather than by near-duplicate cluster made no appreciable difference, which is what the audit predicts: the random scheme split one cluster of the 1276 in the development set, and the grouped scheme none.

Dispersion across the five folds was small. The standard deviation of external balanced accuracy ran from 0.004 for ViT-B/16 AugReg to 0.011 for ConvNeXt-Base.

Resampling by cluster instead of by image, as a check on the dependence the audit might have left behind, gave a mean design effect of 1.02 and moved the confirmatory *p*-values hardly at all, the largest change being from 0.031 to 0.034.

## 4. Discussions

### 4.1. What the Comparison Showed

Six frozen encoders, three convolutional and three attentional, were compared under one head, one input pipeline and one evaluation protocol, and the architectural family did not order them. The best and the worst both belong to the ViT-B/16 family, whose three members differ from one another only in how they were pretrained, and the three convolutional encoders sit between the two. Once the ten confirmatory tests were corrected for multiplicity, the three leading encoders, two attentional and one convolutional, could not be told apart.

Reliability behaved as a separate axis. Calibration was sound in development for all six, yet on the external set, one of them, MAE ViT-B/16, returned probabilities carrying less information than the class prevalences alone, yielding a negative skill score while still classifying more than four images in five correctly.

A control experiment placed a floor under those figures. A thumbnail of sixteen pixels, in which no lesion can survive, recovered two thirds of the foot-and-mouth images and reached a balanced accuracy of 0.527 on the external set, against 0.333 for chance and 0.906 for the best encoder.

The maps offered as explanations withstood randomisation of the head and of the backbone; LeGrad did not, once a single block had been randomised. The ranking of the six survived four variants of the design.

### 4.2. Architecture Does Not Order Transfer

Grouping the six by architecture explains little. The three convolutional encoders span 0.010 in external balanced accuracy, from 0.882 down to 0.872, whereas the three ViT-B/16 encoders span 0.087, from 0.906 down to 0.819. The wider spread belongs to the group whose members share an architecture, a patch size and an input resolution, and differ only in what they were pretrained on and how. Whatever orders these six encoders, it is not the presence or absence of convolution.

The statistical picture agrees. The reference separated from neither ViT-B/16 AugReg nor EfficientNetV2-M on either co-primary metric, so the leading group holds members of both families, and what separates cleanly is the bottom of the ranking, where the encoder that sits last is a transformer.

Two cautions bound this reading. Within the ViT-B/16 triad, the objective and the corpus vary together: CLIP was trained contrastively on a web corpus of four hundred million image–text pairs, AugReg by supervised classification on ImageNet-21k, and MAE by masked reconstruction on ImageNet-1k without labels. Released weights do not let the two factors be pulled apart, so the ordering is attributable to the pretraining recipe as a whole rather than to the objective on its own.

The second caution concerns the regime. Every backbone was frozen and read through a linear head, and that setting is known to be unfavourable to masked autoencoders, whose representations gain substantially once the backbone is fine-tuned [41]. The conclusion therefore holds for transfer without fine-tuning, which is what this study set out to test, and should not be carried over to fine-tuned models.

### 4.3. Discrimination and Calibration Come Apart

Ranking the six by discrimination and ranking them by calibration do not return the same answer, and they part company in an instructive place. The Brier skill score orders the encoders exactly as balanced accuracy does, which is to be expected, since the Brier score responds both to how well the classes are separated and to how well the probabilities are calibrated. The expected calibration error, which responds only to the second, orders them differently: ViT-B/16 AugReg is the best calibrated externally at 0.022, while the encoder with the highest balanced accuracy comes third at 0.036.

Development calibration did not anticipate external calibration either. ResNet50-BiT was the best calibrated of the six in development, at 0.019, and the second worst on the external set, at 0.040.

MAE ViT-B/16 is the extreme case and the clearest one. It reached a balanced accuracy of 0.819, a figure that would pass unremarked in most reports, yet its probabilities carried negative skill against the class prevalences of the same images. A system reporting those numbers to a veterinarian would be wrong about its own certainty while being right about the class more often than not.

The practical consequence is that choosing an encoder on accuracy alone is not enough when what leaves the system is a probability rather than a label [27]. Under this protocol the two properties had to be measured apart, and they returned different winners.

### 4.4. How Much of the Task Needs a Lesion

Sixteen pixels cannot show a vesicle on a dental pad or a nodule on a flank, yet a 4 × 4 thumbnail placed the external images in the right class 0.527 of the time and recovered 0.650 of the foot-and-mouth cases. The floor disposes of the obvious objection: with the training labels shuffled, the same pipeline fell to 0.374, close to the 0.333 of chance, so the descriptors are reading the images and not the procedure.

What they read is the composition of the photograph. Six colour statistics recovered 0.670 of the lumpy skin disease images and barely rose above chance on the foot-and-mouth ones, while a coarse thumbnail reversed the pattern. Colour picks out the class photographed at a distance against pasture and sky; coarse geometry picks out the class photographed as a close-up of a mouth held open by gloved hands. Raising the thumbnail to 16 × 16 added nothing over 8 × 8, so what is exploited is global layout and not detail.

That structure belongs to the source records rather than to the curation applied here. The external foot-and-mouth record is a set of clinical captions naming a body region, whereas the lumpy skin disease record is a folder of 256 × 256 skin patches (Section 2.1.2), so acquisition style covaried with class before a single image was removed.

This is the failure mode described by Geirhos et al., in which a classifier reaches high accuracy on cues that accompany the label without belonging to the disease [28]. The measurements do not establish that the six encoders leaned on those cues; they establish that a large part of the task can be solved with no lesion visible at all. The distance between the thumbnail and the encoders, 0.292 to 0.380 in balanced accuracy, is the most that can be credited to what a lesion adds.

### 4.5. What the Saliency Analysis Supports

The randomisation checks [29] answer one question and leave another untouched. They ask whether a map is a function of the trained model, and the gradient–activation maps passed: destroying the head drove the correlation with the original close to zero, and for two encoders below the level a map of a different image reaches. They do not ask whether the map falls on a lesion.

LeGrad failed the first question in a specific way. Randomising the last block alone changed the predicted class in between a third and two thirds of the images, so the model behind the map was no longer the model under study, and the map nevertheless stayed almost where it was, at correlations above 0.93. Under the full cascade it did decorrelate, so the failure is bounded rather than total; but a map insensitive to the block nearest the output is not reporting on the decision it accompanies. What Figure 5 shows is accordingly the gradient–activation product.

Passing a sanity check is a weak guarantee, and it is the only one available here. No veterinary specialist assessed these maps, so nothing in this study supports the claim that any of them localises a lesion, and the criterion itself has been questioned as sufficient [30]. The maps are offered as a record of what each model responds to rather than as clinical evidence, and they can neither confirm nor contradict the measurement of Section 4.4.

### 4.6. Comparison with Previous Work

Reported figures in the cattle-imaging literature sit high. Studies of lumpy skin disease built on ImageNet-pretrained backbones or on purpose-built networks report accuracies around 90.9% [10,11,12,13], work on foot-and-mouth images is scarcer and combines convolutional with Haralick features [15], and the line that predicts disease from clinical or behavioural variables rather than photographs reports 81% to 98% [16,17,18,19]. The external balanced accuracy obtained here, 0.906, is of similar magnitude, yet the two kinds of numbers are not comparable: balanced accuracy over three classes is not accuracy over two, and the figure here comes from images assembled from other sources and read once, after folds had been drawn so that near-duplicates stayed together.

Two things are missing from that literature, and they are what makes the comparison difficult. Calibration is not reported for cattle imaging, so nothing is known about whether the probabilities such systems emit can be read as probabilities; the question has been put for retinal grading [26] but not here. And no study in this line reports a low-information control, so a published accuracy cannot be divided into what the lesion contributes and what the composition of the photograph contributes. Section 4.4 suggests that division is not a minor one: a balanced accuracy of 0.527 was reached from sixteen pixels, against 0.333 for chance.

None of this shows the published figures to be wrong. It shows that their designs do not permit the distinction, which is the pattern Kapoor and Narayanan document across seventeen fields [20]. A study that evaluates on the collection it was trained on and reports validation accuracy [13] is answering a different question from one that evaluates on a separate collection read once.

On the architectural axis, convolutional networks and vision transformers have been compared on medical images before, with answers that depend on the task [25]. This study adds a case in which the family does not decide the outcome.

### 4.7. Deploying a Frozen Encoder

The protocol is inexpensive. Because the backbones never change, each image passes through an encoder once and the resulting vector is reused, and the whole set of heads reported here was fitted in 6.2 min on a single graphics processor. A veterinary group without access to large computing resources can run this comparison, and can repeat it when a new encoder is released, without training a backbone at any point.

Three of the design choices are worth carrying over. A linear head is preferable to a deeper one: inserting a 512-unit hidden layer raised validation macro-F1 by about 0.02 but pushed the fitted temperatures from below 1.25 up to between 1.51 and 1.83, so the gain in separation was paid for in overconfidence. Calibration is better fitted per class than as a single temperature, which lowered external calibration error for every encoder tested. The encoder should not be picked on discrimination alone, since the three that could not be separated on balanced accuracy do differ on calibration, ViT-B/16 AugReg being the best of them externally at 0.022.

For anyone weighing field use, the shape of the errors matters more than the headline figure. Foot-and-mouth disease was recovered in full by five of the six encoders, so no case in the external set went unflagged; the failures fall on the healthy class, whose recall ranged from 0.585 to 0.819. In screening terms, the system is generous with alarms and reluctant to miss disease. Which of those two errors is tolerable depends on what a positive result sets in motion, since a confirmatory test carries a different cost from a movement restriction.

### 4.8. Limitations

Curation removed 51.3% of the images retrieved, and not evenly: within the Kaggle collection, the healthy class lost 41.7% and the foot-and-mouth class 80.7%. The criteria of Section 2.1.3 act on the identity of an image, on whether it repeats, augments or degrades another, and not on how it was taken, so removing a duplicate leaves one representative of that photograph rather than choosing among photographs. That weakens the possibility that curation shaped the class signal without excluding it, and foot-and-mouth is the class where the surviving fraction is smallest.

Neither collection documents how its images were obtained. There is no collection protocol, no record of who assigned the labels or against what diagnostic criterion, and no animal identifier in either source. Grouping the folds therefore rested on a perceptual-hash proxy for image identity rather than on animal identity, and several photographs of one animal could in principle have been split across folds without being detected.

The label space is narrower than the clinical problem. A model restricted to healthy, foot-and-mouth disease and lumpy skin disease places every image in one of the three, so an animal carrying a condition that resembles either disease, or a condition absent from the training set, receives one of these three labels rather than none. The protocol offers no option of declining to answer.

The acquisition confound of Section 4.4 could only be measured indirectly. An attempt to quantify the fraction of the frame occupied by the animal, using two independent segmenters, was abandoned because they disagreed by as much as 85 percentage points on the same image and reached opposite conclusions about the external set; the extent of an animal is not well defined in a macro close-up. What the low-information descriptors bound is how much of the task survives without a lesion, not how far each encoder relied on that route.

Nothing here evaluates whether a saliency map falls on a lesion. Neither collection carries lesion annotation, and no veterinary specialist assessed the maps, so Section 4.5 speaks to the faithfulness of the method and not to its clinical meaning.

Two encoders were read below the resolution they had been fine-tuned at, ResNet50-BiT at 224 rather than 448 and EfficientNetV2-M at 224 rather than 384, which was the price of giving all six the same input. MAE ViT-B/16 was further disadvantaged by a protocol that never fine-tunes.

Finally, the external evidence rests on a single set of 985 images drawn from two records, of which 103 carry the foot-and-mouth label. Per-class estimates for that class are correspondingly imprecise, and whether the ordering reported here survives a third source, another region or a different photographic practice remains untested.

### 4.9. Future Work

The limitations above point to five things worth doing next, in rough order of how much each would change the conclusions. The first is a collection in which every class is contributed by more than one source, acquired prospectively under a standard protocol, with an animal identifier recorded at capture. That alone would lift the acquisition confound and make a subject-disjoint split possible. The second is lesion annotation traced by veterinary specialists, against which a saliency map can be scored instead of merely inspected. The third is confirmation of the labels by RT-PCR or ELISA, so that a photograph carrying the foot-and-mouth label is known to be foot-and-mouth and not one of the vesicular conditions it resembles. The fourth is a wider label space with a reject option, since a model restricted to three classes must place every image in one of them, including images of a disease it has never been shown. The fifth is a comparison of deployable calibration maps more flexible than a per-class vector, and of fine-tuning as an axis complementary to the frozen regime studied here.

## 5. Conclusions

Under frozen transfer on these two collections, what a pretrained encoder brought to this three-class task was set by its pretraining recipe, not by whether it is convolutional or attentional. The contrast is a controlled one. The best encoder and the worst share an architecture, a patch size and an input resolution, and differ only in what they were trained on and how. Within that group, the objective and the corpus vary together, so the credit belongs to the recipe and not to the objective alone.

Part of the task does not need a lesion. A thumbnail of 64 pixels cannot show a vesicle or a nodule, yet it reached 0.567 balanced accuracy on the external set against 0.333 for chance, and recovered 0.709 of the foot-and-mouth cases. It held all three recalls above chance, so that the figure does not come from a degenerate detector. An accuracy reported on collections of this kind mixes what the lesion contributes with what the composition of the photograph contributes, and the two cannot be told apart without a control.

Discrimination and reliability are not one property. One encoder separated the classes respectably and still returned a Brier skill score of −0.030, which means its probabilities were less informative than the prevalences of the cohort it was scoring.

Three controls follow, and any group can run them on its own data. Fit the same head on thumbnails, colour histograms and permuted labels, and publish that floor beside the headline metric. Report external calibration with a proper scoring rule and its skill score against the prevalences of the target cohort, not with a calibration error alone. Where subject identifiers are absent, as they usually are in public veterinary collections, calibrate the near-duplicate threshold against known reprocessing transformations instead of adopting a published value, and state plainly that the evaluation is not subject-disjoint. These controls cost less to run than the benchmark they audit.

## Figures and Tables

**Table 2:** Discrimination on both sets. Best value in each column in bold.

Encoder	Dev BA	Ext BA	Dev Mac F1	Ext Mac F1	Dev MCC	Ext MCC	Dev AUROC	Ext AUROC
ConvNeXt-Base	0.904	0.878	**0.885**	0.773	**0.837**	0.672	0.982	0.970
ResNet50-BiT	0.887	0.872	0.861	0.745	0.803	0.658	0.973	0.970
EfficientNetV2-M	0.884	0.882	0.867	0.780	0.810	0.682	0.975	0.971
ViT-B/16 AugReg	0.909	0.894	0.885	0.790	0.832	0.701	**0.982**	0.974
CLIP ViT-B/16	**0.913**	**0.906**	0.878	**0.816**	0.821	**0.735**	0.981	**0.978**
MAE ViT-B/16	0.868	0.819	0.834	0.648	0.753	0.542	0.967	0.948

**Table 3:** Calibration on both sets, after temperature scaling. Best value in each column in bold. T is the mean fitted temperature.

Encoder	Dev ECE ↓	Ext ECE ↓	Dev Brier ↓	Ext Brier ↓	Dev BSS	Ext BSS	T
ConvNeXt-Base	0.033	0.033	**0.139**	0.281	**0.747**	0.370	1.045
ResNet50-BiT	**0.019**	0.040	0.173	0.307	0.686	0.313	1.243
EfficientNetV2-M	0.049	0.039	0.171	0.255	0.689	0.429	1.218
ViT-B/16 AugReg	0.025	**0.022**	0.144	0.253	0.739	0.433	1.049
CLIP ViT-B/16	0.032	0.036	0.166	**0.233**	0.699	**0.478**	1.061
MAE ViT-B/16	0.037	0.101	0.209	0.460	0.622	−0.030	1.184

The downward arrow indicates that lower values are better; for the Brier skill score (BSS), higher values are better.

**Table 4:** Confirmatory comparisons of CLIP ViT-B/16, the reference encoder, against each of the other five on the external set. Δ is the reference minus the comparator, so a positive value favours the reference. Intervals are percentile bootstrap over 10,000 paired resamples; *p*-values are corrected by the Holm procedure across the ten tests. Values in bold are significant at 0.05.

Metric	Comparator	∆	95% CI	*p* (Holm)
Balanced accuracy	ConvNeXt-Base	+0.028	+0.009 to +0.049	**0.0300**
Balanced accuracy	ResNet50-BiT	+0.034	+0.015 to +0.055	**0.0042**
Balanced accuracy	EfficientNetV2-M	+0.024	+0.002 to +0.047	0.094
Balanced accuracy	ViT-B/16 AugReg	+0.012	−0.006 to +0.031	0.185
Balanced accuracy	MAE ViT-B/16	+0.088	+0.067 to +0.108	**0.0020**
Macro-F1	ConvNeXt-Base	+0.043	+0.016 to +0.071	**0.0108**
Macro-F1	ResNet50-BiT	+0.070	+0.042 to +0.099	**0.0020**
Macro-F1	EfficientNetV2-M	+0.036	+0.007 to +0.065	0.062
Macro-F1	ViT-B/16 AugReg	+0.026	−0.002 to +0.054	0.137
Macro-F1	MAE ViT-B/16	+0.167	+0.139 to +0.196	**0.0020**

## Data Availability

All data analysed in this study are publicly available from third-party repositories; no new data were generated. The development set was obtained from the Kaggle dataset “Cattle diseases datasets” by Devang (devang03mgr), available at https://www.kaggle.com/datasets/devang03mgr/cattle-diseases-datasets (accessed on 3 September 2026) [31]; it comprises 3244 images, of which 1277 were retained after the curation described in Section 2.1.3. The external set was merged from two Zenodo records, “LumpySkinDisease_DataHub” by Branda, F. (2024), available at https://zenodo.org/records/11003967 (accessed on 3 September 2026) [32], and “FMD Cattle” by Osborn, A. (2023), available at https://zenodo.org/records/7779246 (accessed on 3 September 2026) [33]; together they comprise 1399 images, of which 985 were retained. Further inquiries can be directed to the corresponding author.
